# Epileptic High-Frequency Oscillations in Intracranial EEG Are Not Confounded by Cognitive Tasks

**DOI:** 10.3389/fnhum.2021.613125

**Published:** 2021-02-24

**Authors:** Ece Boran, Lennart Stieglitz, Johannes Sarnthein

**Affiliations:** ^1^Klinik für Neurochirurgie, Universitäts Spital und Universität Zürich, Zurich, Switzerland; ^2^Neuroscience Center Zurich, University of Zurich and ETH Zurich, Zurich, Switzerland

**Keywords:** epilepsy surgery, seizure onset zone, epileptogenic zone, medial temporal lobe, working memory, emotional processing, hippocampus, amygdala

## Abstract

**Rationale**: High-frequency oscillations (HFOs) in intracranial EEG (iEEG) are used to delineate the epileptogenic zone during presurgical diagnostic assessment in patients with epilepsy. HFOs are historically divided into ripples (80–250 Hz), fast ripples (FR, >250 Hz), and their co-occurrence (FRandR). In a previous study, we had validated the rate of FRandRs during deep sleep to predict seizure outcome. Here, we ask whether epileptic FRandRs might be confounded by physiological FRandRs that are unrelated to epilepsy.

**Methods**: We recorded iEEG in the medial temporal lobe MTL (hippocampus, entorhinal cortex, and amygdala) in 17 patients while they performed cognitive tasks. The three cognitive tasks addressed verbal working memory, visual working memory, and emotional processing. In our previous studies, these tasks activated the MTL. We re-analyzed the data of these studies with the automated detector that focuses on the co-occurrence of ripples and FRs (FRandR).

**Results**: For each task, we identified those channels in which the HFO rate was modulated during the task condition compared to the control condition. However, the number of these channels did not exceed the chance level. Interestingly, even during wakefulness, the HFO rate was higher for channels within the seizure onset zone (SOZ) than for channels outside the SOZ.

**Conclusion**: Our prospective definition of an epileptic HFO, the FRandR, is not confounded by physiological HFOs that might be elicited by our cognitive tasks. This is reassuring for the clinical use of FRandR as a biomarker of the EZ.

## Introduction

When considering epilepsy surgery, the recording of intracranial EEG (iEEG) is a standard procedure to identify the seizure onset zone (SOZ; Jobst et al., 2020). There is accumulating evidence that high-frequency oscillations (HFOs > 80 Hz) in the iEEG are a reliable biomarker of epileptogenic tissue, bearing the potential to guide the surgical treatment of drug-resistant focal epilepsy (Jacobs et al., 2009; Fedele et al., 2016, 2017a, 2019; van ’t Klooster et al., 2017; Jacobs and Zijlmans, 2020; Chen et al., 2021).

First reports in groups of patients showed that HFOs have higher rates in electrode contacts within the SOZ than outside the SOZ (non-SOZ; Jacobs et al., 2009). In individual patients, the aim is to delineate the epileptogenic zone (EZ). The EZ is defined as the area of the cortex whose resection leads to seizure freedom. HFOs have been shown to indicate the EZ both in intraoperative ECoG (Fedele et al., 2016, 2017b; van ’t Klooster et al., 2017; Weiss et al., 2018; Boran et al., 2019c) and in presurgical iEEG recordings (Akiyama et al., 2011; Fedele et al., 2017a) while the results of a clinical trial are still pending (van ’t Klooster et al., 2015). Furthermore, the HFO rate in surface EEG mirrors epilepsy severity (Boran et al., 2019d; Fan et al., 2020; Klotz et al., 2021).

HFOs are historically divided into ripples (80–250 Hz), fast ripples (FRs, >250 Hz), and their co-occurrence (FRandR). HFOs were first detected in the medial temporal lobe (MTL) of rodents, independent of epilepsy but associated with cognitive function (Buzsáki, 2006). Furthermore, HFOs occur in central and occipital brain regions without a relationship to epilepsy (Frauscher et al., 2018). These HFOs were therefore termed physiological HFOs. Unfortunately, different studies use the term “HFO” for different phenomena (Noorlag et al., 2019). The distinction between a physiological HFO and an epileptic HFO, which indicates the EZ, is a matter of ongoing research (Cimbalnik et al., 2018, 2020; Frauscher et al., 2018; Weiss et al., 2019, 2020; Arnulfo et al., 2020; Gliske et al., 2020; Pail et al., 2020). Can an epileptic HFO be confounded with a physiological HFO? The distinction has important implications: Confounding might entrain an erroneous delineation of the EZ and, in consequence, suboptimal surgical decisions.

To improve the clinical applicability of HFO, ideas on good practice have been summarized (Fedele et al., 2019; Chen et al., 2021). First, an epileptic HFO must aim to delineate the EZ and be validated against seizure outcome. Second, there must be a prospective definition of what should be marked as an epileptic HFO, as can be achieved by an automated detector (Fedele et al., 2016, 2017a; Weiss et al., 2018; Boran et al., 2019c, d; Nariai et al., 2019). Third, the data epochs should be carefully selected. In clinical research, presurgical iEEG data is usually selected from artifact-free epochs during deep sleep.

The detection of HFOs has been facilitated by automated or semi-automated detection algorithms (Remakanthakurup Sindhu et al., 2020. Of note, the vast literature on detection algorithms reflects the vast variety of definitions of what is considered to be an HFO. Here we apply a fully automated definition of HFOs, which we previously optimized on visual markings in a dataset of the Montreal Neurological Institute (Burnos et al., 2016b) and then validated on independently recorded data from Zurich (Fedele et al., 2017a). In that study, FRandRs turned out to predict seizure freedom after resective epilepsy surgery with the highest accuracy (Fedele et al., 2017a). In a further study on an independent dataset from Geneva, we again found high accuracy for outcome prediction (Dimakopoulos et al., 2020). From these studies, we deduce that FRandR are the best definition of an epileptic HFO in iEEG and therefore focus our analysis on FRandR.

Furthermore, we define as a physiological HFO an oscillation whose occurrence does not reflect the pathology and that may be induced by a cognitive task (Axmacher et al., 2008; Kucewicz et al., 2014; Arnulfo et al., 2020).

In the present study, we address the distinction between epileptic and physiological HFOs in the human MTL. For the selection of data, we build on earlier studies where we asked patients to perform cognitive tasks while we recorded iEEG. In these earlier studies, we recorded and associated the firing of single neurons with task performance, thereby confirming that the tasks were indeed activating regions of the MTL in the patients of this study (Boran et al., 2019a, 2020b). The datasets are published for re-analysis (Boran et al., 2019b, 2020a; Dimakopoulos et al., 2020; Fedele et al., 2020a, 2021).

We hypothesized that our prospective definition of an epileptic FRandR (Fedele et al., 2017a) is not confounded by physiological HFOs in the MTL. As our null hypothesis, the rate of FRandRs should be unaffected by the cognitive processing during task performance. We found a null result, i.e., cognitive processing did not modulate the FRandR rate greater than expected by chance.

## Materials and Methods

### Subjects

The subjects were patients with epilepsy (17 subjects, age 18–56 years, 10 males, Table 1) that had iEEG electrodes implanted in their MTL during the presurgical diagnostic workup. All subjects had a normal or corrected-to-normal vision and were right-handed as confirmed by neurophysiological testing. Each subject performed at least one of the cognitive tasks.

**Table 1:** Subject characteristics. Subjects were implanted in the medial temporal lobe (MTL) and performed at least one cognitive task.

Subject number	Age	Sex	Pathology	Electrodes	SOZ	Verbal working memory	Visual working memory	Fearful number faces
1	31	Male	Hippocampal sclerosis	AHL, AHR, AL, AR, ECL, ECR, PHL, PHR	AR, ECR	x	x	x
2	18	Female	Hippocampal sclerosis	AHL, AHR, AL, ECL, PHL	AHL, AL, ECL, PHL	x	x	x
3	39	Male	Gliosis	AHL, AHR, AL, AR, ECL, ECR, PHL, PHR	AHR, PHR	x	x	x
4	28	Male	Brain contusion	AHL, AHR, AL, AR, ECL, ECR, PHL, PHR	AHL, AHR, PHL, PHR	x	x	-
5	47	Male	Hippocampal sclerosis	AHL, AHR, AL, AR, ECL, ECR, PHL, PHR	AHR, PHR	x	-	x
6	19	Female	Hippocampal sclerosis	AHL, AHR, AL, AR, ECL, ECR, PHL, PHR	AR, ECR	x	-	-
7	24	Female	Xanthoastrozytoma WHO II	AHL, AL, ECL, LR, PHL, PHR	LR	x	-	-
8	56	Female	Hippocampal sclerosis	AHL, AHR, AL, AR, ECL, ECR, PHL, PHR	ECR	x	-	-
9	20	Female	Focal cortical dysplasia	AHL, AL, DRR, PHR	DRR	x	-	-
10	31	Female	Hippocampal sclerosis	AHR, ECR, PHR	AHR, PHR	-	x	-
11	35	Male	Unknown	AHL, AHR, AL, AR, ECL, ECR, PHL	AHL	-	x	-
12	20	Male	Focal cortical dysplasia	AHL, AHR, PHL, PHR		-	x	-
13	19	Male	Unknown	AHR, PHL, PHR	PHL	-	x	-
14	51	Female	Hippocampal sclerosis	AHL, AHR, AL, AR, ECL, ECR, PHL, PHR	AHL, PHL	-	x	-
15	21	Male	Hippocampal sclerosis	AL		-	-	x
16	22	Male	Hippocampal sclerosis	AL, AR	AR	-	-	x
17	21	Male	Hippocampal sclerosis	AL, AR		-	-	x

*L, left; R, right; AH, hippocampal head; PH, hippocampal body; EC, entorhinal cortex; A, amygdala; DRR, LR: lesions, AIR, insular gyrus right; FR, frontal right; SOZ, seizure onset zone; (x) subject performed the task; (−) subject did not perform the task. The FR and R rates in SOZ channels and nonSOZ channels differed significantly in only 8/17 subjects (Wilcoxon rank-sum test *p* < 0.05)*.

### Data Acquisition and Selection

Depth electrodes (1.3 mm diameter, eight contacts of 1.6 mm length, and spacing between contact centers 3 mm or 5 mm; Ad-Tech^1^, Racine, WI, UDA) were stereotactically implanted into the amygdala, hippocampus, and entorhinal cortex bilaterally (Table 1). iEEG was recorded against a common reference at a sampling frequency of 4,000 Hz with the ATLAS recording system (0.5–1,000 Hz pass-band, Neuralynx, www.neuralynx.com). For HFO analysis, iEEG signals were resampled at 2,000 Hz and transformed to a bipolar montage. We removed channels with high noise levels or many artifacts and invalid trials.

In parallel to the iEEG data presented here, we used microelectrodes and high-resolution equipment to record neuronal firing, which has been reported previously (Fedele et al., 2017a; Boran et al., 2019a, 2020b).

### Electrode Localization

**Figure 1:**
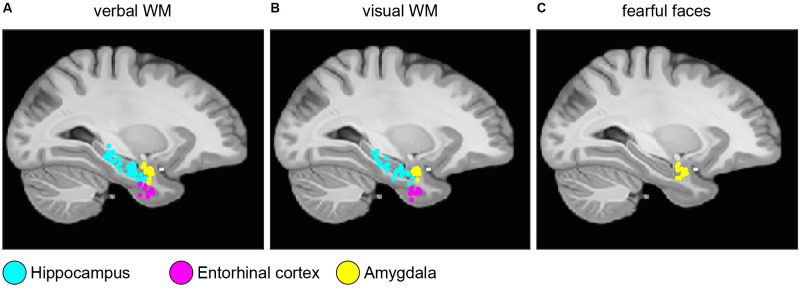
Electrode localization. Anatomical locations of the tips of the depth electrodes in Montreal Neurological Institute’s MNI152 space (Methods) for **(A)** verbal working memory task; **(B)** visual working memory task; **(C)** fearful faces task. Locations are projected on the parasagittal plane *x* = −25.2 mm and are color-coded (cyan, hippocampus; magenta, entorhinal cortex; and yellow, amygdala).

Electrode localization and clinical data were taken from the published datasets (Boran et al., 2019b, 2020a; Fedele et al., 2020a, 2021). In brief, the patients were implanted with iEEG electrodes in MTL at Universitätsspital Zürich. Electrodes were localized using postimplantation CT scans and postimplantation structural T1-weighted MRI scans. For each subject, the CT scan was registered to the postimplantation scan as implemented in FieldTrip (Oostenveld et al., 2011; Stolk et al., 2018). In the coregistered CT-MR images, the electrode contacts were visually marked. The contact positions were normalized to the MNI space and assigned to a brain region using the Brainnetome Atlas (Fan et al., 2016). Also, depth electrode positions were verified by the neurosurgeon (LS) after merging preoperative MRI with postimplantation CT images of each subject in the plane along the electrode (iPlan Stereotaxy 3.0, Brainlab, München, Germany). We grouped electrodes according to their anatomical region (Hipp: hippocampus, Ent: entorhinal cortex, Amg: amygdala) and whether they were recorded within the SOZ or outside the SOZ. Figure 1 shows the localization of the electrode tips projected on a parasagittal plane (MNI space *x* = −25.2 mm).

### Clinical Data and SOZ

Patients underwent a presurgical diagnostic workup at Schweizerische Epilepsie-Klinik. The clinical information was taken from the hospital patient records. The SOZ was defined by experienced epileptologists independent of the studies.

### Tasks Activating the MTL Guided iEEG Data Selection

Our selection of iEEG data was guided by whether we had found neuronal firing in the same subjects that were associated with task performance (Boran et al., 2019a, 2020b; Fedele et al., 2020b). Our previous analysis of neuronal firing in the MTL served to characterize task demand and to predict subject behavior, thus demonstrating the involvement of MTL in cognitive task performance. Only then we could be assured that this structure of MTL in this subject was actually engaged in task processing.

#### Verbal Working Memory Task

**Figure 2:**
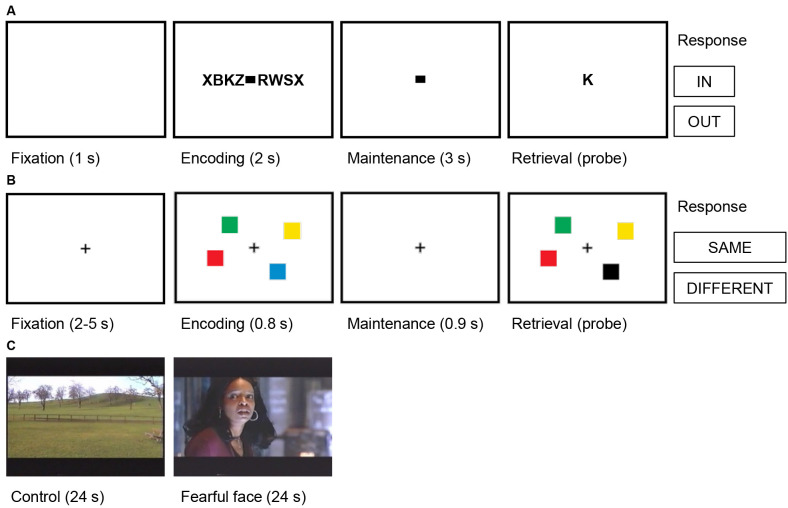
Trial structures for the cognitive tasks. **(A)** Verbal working memory task. In this task, sets of consonants were presented and had to be memorized. Each trial (50 trials per session) started with a fixation period (1 s), followed by the presentation of a letter string (encoding, 2 s). The number of letters presented determined WM workload (task condition/high workload: six or eight letters; control condition/low workload: four letters). The encoding period was followed by a delay (maintenance, 3 s). After the delay, a probe letter was shown, and subjects indicated whether the probe was presented during the encoding period (In/Out). **(B)** Visual working memory task. In this task, visual working memory was examined using a change detection task. In each trial (192 trials per session), a fixation period (2–5 s) was followed by the presentation of the memory array of colored squares (encoding, 0.8 s). The number of squares determined WM workload (task condition/high workload: four or six squares; control condition/low workload: one or two squares). The encoding period was followed by a delay (maintenance 0.9 s). After the delay, a probe array was shown, and subjects indicated whether the probe array differed from the memory array (Same/Different). **(C)** Fearful faces. In this task, amygdalar response to fear was examined using fearful faces. Alternating blocks of fearful faces (task condition, eight trials) and neutral landscapes (control condition, nine trials) were shown. Each block lasted 24 s and consisted of short video clips of 2–3 s. Video clips of fearful faces were extracted from thriller and horror movies and contained faces of actors showing fear. In each trial, the block was preceded by a repeated baseline of 2 s of a neutral landscape.

To activate verbal working memory, we used a modified Sternberg task where the subject had to memorize a string of letters (Figure 2A; Boran et al., 2019a). The number of letters in the string determined the working memory load (low workload: four letters; high workload: six or eight letters; 50 trials per session; 36 sessions in total). The mean duration of recording in each subject was 23.3 min. The behavioral results of the subjects were as expected from a working memory task: the rate of correct responses decreased with set size from 4 (98.5% correct responses) to set sizes of 6 (90.5%) and 8 (84.7%). The mean response time for the correct trials (1630 trials) increased with workload (48 ms per item). We analyzed a total of 773 MTL channels from nine subjects for this task (Table 1).

We have reported earlier (Boran et al., 2019a) that for the same task in the same subjects, we found neurons in the MTL that fired persistently during the maintenance period. Some of these neurons increased their firing rate for a high workload. We could also decode the workload of single trials from the neuronal population firing in the MTL. As a robust finding, hippocampal iEEG activity and hippocampal-cortical synchronization was high for trials with high workload and not for trials with four letters. Therefore, trials with four letters were taken as the control condition.

#### Visual Working Memory Task

To activate visual working memory, we used a change detection task where the subject had to memorize an array of colored squares (Figure 2B; Boran et al., 2020b). The number of squares determined the working memory load (low workload: one or two squares; high workload: four or six squares; 192 trials per session). For each subject, the duration of the session was 11.5 min. The rate of correct responses decreased with set size from a set size of 1 (98% correct responses) to 2 (99%), 4 (88%), and 6 (73%). The mean response time for the correct trials (2,678 trials) increased with set size (118 ms/item). We analyzed a total of 178 MTL channels from nine subjects for this task (Table 1).

We have reported earlier (Boran et al., 2020b) that for the same task in the same subjects, we found neurons in the MTL that fired persistently and increased their firing rate for trials with a high workload during the maintenance period. Neuronal population firing in the MTL during maintenance distinguished workload and we could decode workload of single trials. Therefore, trials with one or two squares were taken as the control condition.

#### Fearful Faces Task

To activate the amygdala during emotional processing, we presented fearful faces as dynamic visual stimuli (Figure 2C; Fedele et al., 2020b). For trials of the aversive condition (eight trials), a 24 s block of short video clips (2–3 s) of fearful faces were shown. Video clips of fearful faces were extracted from thriller and horror movies and contained faces of actors showing fear. For trials of the control condition (nine trials, 24 s each), the video clips were from neutral landscapes. Each trial started with a repeated baseline of a 2 s video of a neutral landscape and there were seven sessions in total. For each subject, the duration of the task was 7 min.

We have reported earlier (Fedele et al., 2020b) that for the same task in the same subjects, for the aversive compared to the control condition, amygdalar high gamma power (>60 Hz) increased during the first 2 s and delta power (1–4 Hz) decreased for up to 18 s. Also, neuronal firing increased during the aversive condition. The high correlation of these measures with the BOLD response in the same subjects (Schacher et al., 2006) points to high gamma, delta, and neuronal firing being the electrophysiological counterparts to the observed increase in BOLD response during emotional processing in the amygdala. Since the task was designed to activate the amygdala (Schacher et al., 2006) and we found task-related neuronal firing only in the amygdala of these subjects (Fedele et al., 2020b), we here report only iEEG data from the 12 amygdalar channels of these subjects (Table 1).

### Automated HFO Detection

**Figure 3:**
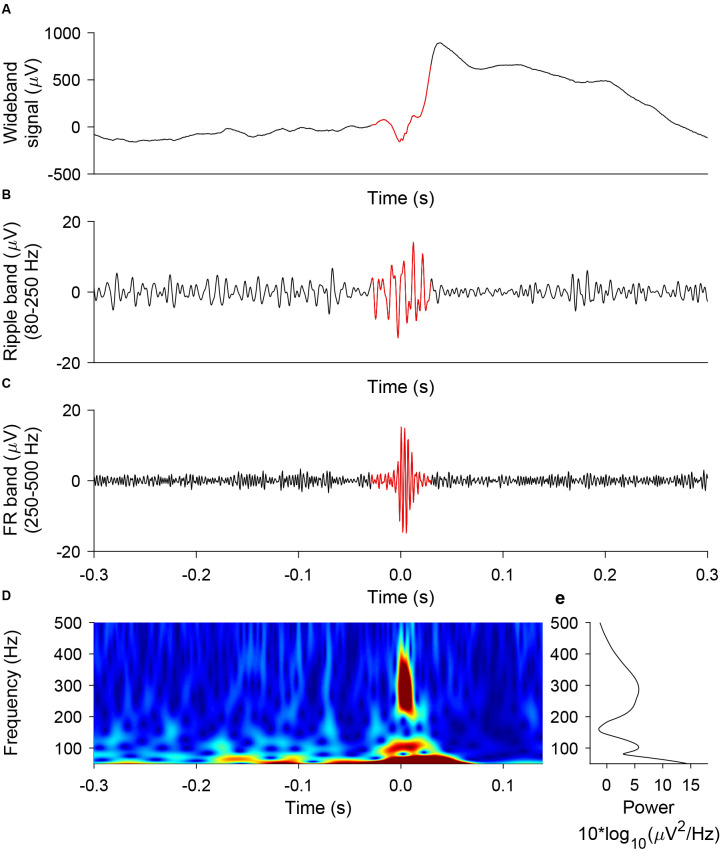
Representative example of ripple, FR, FRandR. A ripple co-occurring with a fast ripple (FRandR) is shown **(A)** in the wideband signal, **(B)** the signal filtered in the ripple band (80–250 Hz), and **(C)** the signal filtered in the FR band (250–500 Hz). **(D)** The instantaneous frequency spectrum is smooth and does not allow a distinction between ripples and FR, in agreement with our earlier finding (Fedele et al., 2017a).

We used the prospective HFO detector previously validated to predict seizure outcome from iEEG recorded during intervals of NREM sleep (Fedele et al., 2017a). The detector captures the morphology of an HFO and was developed on data from the Montreal Neurological Institute (Burnos et al., 2016b). In brief, the detector has a baseline detection stage and an HFO detection stage that are performed separately for ripples and FRs (Burnos et al., 2016b). In the baseline detection stage, the segments of the signal corresponding to the baseline are determined using Stockwell entropy. The amplitude threshold is defined using these segments. In the HFO detection stage, events, where the filtered signal exceeded the amplitude threshold for at least 20 ms, were defined as ripples. Similarly, events, where the filtered signal exceeded the amplitude threshold for at least 10 ms, were defined as FR. Furthermore, we defined a FRandR as the co-occurrence of a ripple and an FR (Fedele et al., 2017a). Figure 3 shows a representative example of a ripple, an FR, and the corresponding FRandR.

Similar to HFO detection during intervals of NREM sleep, HFOs were detected on the continuous data recorded while the subject performed the tasks. We used the timestamps of the HFOs to assign them to trials of task or control conditions. We computed the rate of ripples, FRs, and FRandRs during the cognitive tasks for each channel separately. We use the term HFO to comprise all three types of HFO (ripple, FR, and FRandR).

### HFO Rate Comparison Between Task and Control Conditions

We tested whether the HFO rates were modulated during the task condition as compared to the control condition. The choice of control condition was based on the design of the tasks and our previous reports of single neuron firing in the same patients (Boran et al., 2019a, 2020b; Fedele et al., 2020b). To assure that subjects were actually engaged in the task, we only used trials where the subject responded correctly.

For the verbal working memory task (Boran et al., 2019a), we compared the HFO rate during maintenance for low workload trials (set size 4) and high workload trials (set size 6 or 8) within each anatomical region.

For the visual working memory task (Boran et al., 2020b), we compared the HFO rate during maintenance for low workload trials (set size 1 or 2) and high workload trials (set size 4 or 6) within each anatomical region.

For the fearful faces task (Fedele et al., 2020b), we compared the HFO rate during the presentation of stimuli for trials with fearful faces (aversive condition) and trials with neutral landscapes (neutral condition).

### Statistics

To assess the significance of the difference of HFO rates across task conditions, we used the Wilcoxon rank-sum test. Next, we determined the number of channels where the HFO rate increased or decreased significantly (Wilcoxon rank-sum test). Furthermore, to assess the significance of the number of channels showing any effect, we used a permutation test with scrambled labels: we created a null distribution estimated from *n* > 200 permutations on data with scrambled labels. For the permutation test, the iEEG of each task condition was considered as 1 bin; we did not split the iEEG further. The minimum *p-value* is limited by the number of permutations as *p* = 1/(number of permutations + 1). Reported *p-values* were based on the percentage of values in the empirically estimated null distribution that was exceeded by the observed value. For all tasks and channels, we performed the analysis separately for all types of HFOs (ripples, FRs and FRandRs).

## Results

### HFO Rate Does Not Differ Between Task and Control Condition

**Figure 4:**
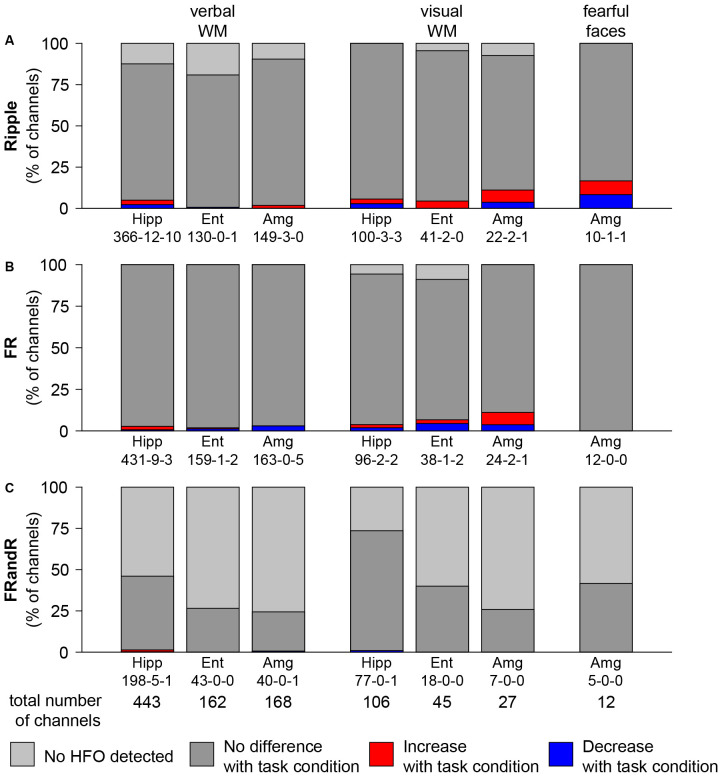
HFO rate does not change with task condition. Percentage of channels with changes in **(A)** ripple, **(B)** FR, and **(C)** FRandR rates with task conditions during cognitive tasks. Left: verbal working memory task. Channels with modulation of HFO rates for the task condition (six or eight letters) vs. the control condition (four letters). From a total of 443 channels analyzed in the hippocampus, in 198 there were FRandRs detected. In five of these channels, the FRandR rate increased, and in one channel FR, and R rate decreased (198-5-1). Middle: visual working memory task. Channels with modulation of HFO rates for the task condition (four or six squares) vs. the control condition (one or two squares). Right: fearful faces task. Channels with modulation of HFO rates for fearful faces condition vs. control condition. The percentage of channels with increase or decrease with task conditions do not reach significance for any HFO type or task (permutation test with scrambled labels).

To test our primary hypothesis, we identified channels where the HFO rate was modulated by the task condition compared to the control condition. The median HFO rate over all the tasks was 4.14, 2.38, and 0.07 events/min for ripples, FRs, and FRandRs, respectively. The absolute and relative numbers of channels where task condition changed the HFO rate either up or down is given in (Figure 4).

For the verbal working memory task, ripple rates increased or decreased for the task condition (six or eight letters) compared to the control condition (low workload trials with four letters) during maintenance for a few channels. Figure 4A shows the number of channels for all subjects that show an increase (red bars) or decrease (blue bars) in ripple rate with the workload for each anatomical region. For hippocampus, entorhinal cortex, and amygdala, 22, 1 and 3 channels had ripple rates that differed with workload (*p* <0.05, Wilcoxon rank-sum test for individual channels). However, there is a large number of channels in each MTL region. We, therefore, tested the significance of the number of channels that show any effect by comparing against a random distribution. The number of channels with ripple rates that were modulated by the task for any MTL region was not significant (*p* = 0.5150, *p* = 1.0000, and *p* = 0.9750, permutation test against scrambled labels). Likewise, several channels show FR (Figure 4B) and FRandR (Figure 4C) rates that are modulated by the task. Similarly, these numbers did not exceed the chance level for any region (*p* >0.05, permutation test against scrambled labels).

For the visual working memory task, we also found channels with modulation in HFO rate during the task (Figure 4; task condition, four or six squares; control condition, one or two squares). With the same statistical approach as above, the number of these channels did not exceed the chance level for any MTL region (for ripples, *p* = 0.3450, *p* = 0.6650, and *p* = 0.1750, permutation test against scrambled labels).

During the presentation of the fearful faces, there was one channel where ripple rate increased or decreased for the task condition, respectively. Similar to the working memory tasks, the number of channels that showed such effect was not significant (*p* = 0.1000, permutation test against scrambled labels).

There was no significant difference between channels recorded from the left or the right hemisphere of the brain. There was no significant association between channels in the five subjects that performed more than one task.

Overall, the number of channels in the MTL with HFO rates that were modulated by the task was not greater than expected by chance.

### HFO Rate During Task Performance Differs Between SOZ and Non-SOZ

In addition to our primary hypothesis, we tested whether HFO rates were higher within the SOZ than outside the SOZ.

**Figure 5:**
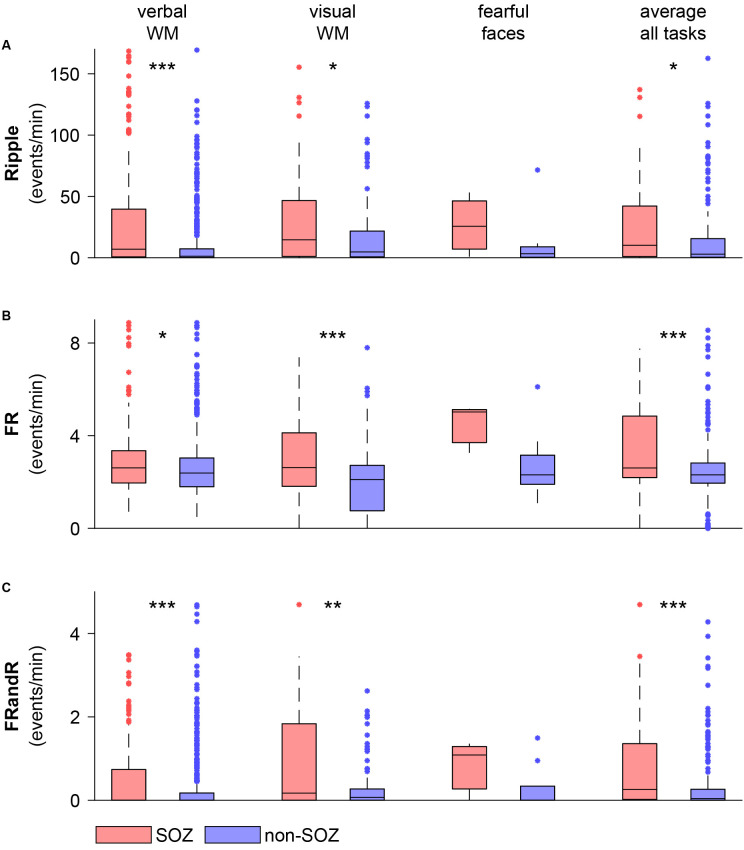
HFO rate is higher within the SOZ than outside the SOZ during task performance. **(A)** Ripple, **(B)** FR, and **(C)** FRandR rates for channels within and outside the SOZ for each cognitive task (verbal WM: verbal working memory task, visual WM: visual working memory task, fearful faces: fearful faces task). The rightmost columns show average HFO rates over all the tasks and all sessions for each channel within and outside the SOZ. Over the patient group, HFO rates for channels within the SOZ are higher than for non-SOZ channels for all HFO types for the memory tasks and the average over all the tasks (*p* <0.05, Wilcoxon rank-sum test). ***p < 0.001, **p < 0.01, *p < 0.05.

For the verbal working memory task, the HFO rate in the SOZ (213 channels) exceeded the HFO rate outside the SOZ (560 channels) for ripples (Figure 5; *p* = 1.486 × 10^−9^, Wilcoxon rank-sum test), FRs (*p* = 0.0128, Wilcoxon rank-sum test) and for FRandRs (*p* = 2.207 × 10^−6^, Wilcoxon rank-sum test).

Similarly, for the visual working memory task, HFO rates were higher within the SOZ (56 channels) than outside the SOZ (122 channels) for ripples (*p* = 0.0374, Wilcoxon rank-sum test), FRs (*p* = 0.0008, Wilcoxon rank-sum test) and for FRandRs (*p* = 0.0044, Wilcoxon rank-sum test).

For the fearful faces task, HFO rates were higher within the SOZ (three channels) than outside the SOZ (nine channels). Due to the small number of channels, this difference did not reach significance for ripples (*p* = 0.3727, Wilcoxon rank-sum test), FRs (*p* = 0.1000, Wilcoxon rank-sum test) and for FRandRs (*p* = 0.3455, Wilcoxon rank-sum test).

For individual subjects, HFO rates average over tasks were higher within the SOZ than outside the SOZ for FRand R in only 8/17 subjects (FR 6/17; ripple 7/17). When averaging over all subjects and tasks, HFO rates were higher within the SOZ (77 channels) than outside the SOZ (197 channels) for ripples (*p* = 0.0114, Wilcoxon rank-sum test), FRs (*p* = 0.0008, Wilcoxon rank-sum test) and FRandRs (*p* = 0.0001, Wilcoxon rank-sum test).

## Discussion

When comparing HFO rate between task and control condition, HFO rates did not change greater than expected by chance. This favors our main hypothesis: there was no indication that the HFOs as prospectively defined in (Fedele et al., 2017a) were confounded by physiological HFOs. As an additional finding on the group level, HFO detected during active wakefulness were found to be more abundant in the SOZ and therefore also reflected pathology.

### Methodological Considerations

Our primary methodological consideration is the definition of an HFO. We used our automated HFO detector which was designed to analyze long-term iEEG recordings during NREM sleep (Burnos et al., 2016b). The detection algorithm has been validated to predict seizure outcome after resective epilepsy surgery with good accuracy (Burnos et al., 2016b; Fedele et al., 2017a). Here we used this detector “off-label” on awake subjects performing cognitive tasks.

We based our prospective definition of a clinically relevant HFO on the co-occurrence of a ripple and a fast ripple (FRandR), where the majority of FRandR show an instantaneous frequency spectrum that does not distinguish between ripples and FR (Figure 3; Fedele et al., 2017a). We thus ignored the traditional distinction between ripples (80–250 Hz) and FR (250–500 Hz; Lévesque et al., 2018; Chen et al., 2021). As expected, the FRandR rate was much lower than the rates of ripples and FR separately.

In our HFO analysis, we used a bipolar montage, i.e., we subtracted the signal from two adjacent electrode contacts and considered the difference as a recording channel. The subtraction eliminates spatially extended background activity and artifacts, above all the line hum and its harmonics. Because of the small amplitude of HFOs (Fedele et al., 2017b), this subtraction was mandatory in all the datasets from several institutions that we analyzed (Burnos et al., 2016b; Fedele et al., 2016, 2017b; Dimakopoulos et al., 2020). Furthermore, the bipolar montage affects our certainty concerning the spatial origin of an HFO. On the mm scale, there is evidence that HFOs are generated by a tissue area in the millimeter range (Boran et al., 2019c; Zweiphenning et al., 2020). In principle, a FRandR might result from the superposition of a ripple at one contact and an FR at the other contact of a recording channel (spacing ≤5 mm) (Zaveri et al., 2006), if one would assume that FRandR were composed of distinct entities. On a larger scale, the bipolar montage ensures that the HFO is generated in the vicinity of the two contacts and not somewhere between one contact and the recording reference (spacing ~5 cm). This agrees with the clinical standard where the SOZ is detected in a bipolar montage.

Finally, we addressed the problem of multiple comparisons. A large number of channels entered the analysis and a significant modulation of some channel’s HFO rate would be expected simply by chance as a spurious effect. We, therefore, applied computational statistics to calculate the statistical significance of the percentage of channels where the cognitive tasks modulated HFO rate either up or down. We found that this number of channels was not greater than expected by chance.

### Physiological and Epileptic HFOs

Spontaneous physiological HFOs were first described in the hippocampus (Buzsáki, 2006). In neocortical areas, somatosensory stimulation elicited physiological HFOs (Burnos et al., 2016a; Fedele et al., 2017c). Spontaneous physiological HFO in the neocortex were mainly observed in central and occipital areas (Nagasawa et al., 2012; Frauscher et al., 2018). An attempt to distinguish individual physiological and epileptic HFOs by their morphology proved unsuccessful (Burnos et al., 2016b). For clinical applications of HFOs, distinguishing physiological and epileptic HFOs is a major concern. Including physiological HFOs in the analysis may lead to an erroneous estimation of the EZ, resulting in suboptimal surgical decisions and suboptimal clinical outcomes (Chen et al., 2021).

### FRandR Rate Was Not Modulated by Task Performance

As our main result, the FRandR rate during task performance did not change greater than the chance level, i.e., a null result (Figure 4). While we found the same null result for all three types of HFO (ripple, FR, and FRandR), we focus our discussion on FRandR because FRandR had the highest accuracy in predicting seizure outcome after resective epilepsy surgery (Fedele et al., 2017a). From this null result, we conclude that FRandRs are not confounded by task-related HFOs. We discuss this conclusion because of the following questions.

Do these subjects perform these tasks without activating the brain regions where we record from? To prove that the recordings are indeed from activated brain areas, we have selected iEEG data from subjects where we had reported task-related neuronal firing in the MTL of the same subjects (Boran et al., 2019a, 2020b; Fedele et al., 2020b). This assured us that these subjects activated their MTL to perform the tasks.

Are FRandR valid biomarkers for epileptogenic tissue? In our search for an automated definition of an epileptic HFO, we aimed to predict the seizure outcome after resective epilepsy surgery (seizure-free vs. not seizure-free postoperatively; Fedele et al., 2019). Here, FRandR turned out to have the highest accuracy (Fedele et al., 2017a). Our approach is different from other approaches in the literature (Chen et al., 2021). For example, several studies in humans define the distinction of physiological and epileptic HFOs by assuming that an HFO that occurs in the SOZ is epileptic, while an HFO outside the SOZ or in the sensory or motor cortices is physiological (Cimbalnik et al., 2018; Frauscher et al., 2018; Weiss et al., 2019, 2020; Gliske et al., 2020; Remakanthakurup Sindhu et al., 2020). Similarly, we found increased FRandR activity in the SOZ (Figure 5). Thus, we deduce from the results presented in Figure 5 and more comprehensive results presented earlier (Fedele et al., 2017a; Dimakopoulos et al., 2020), that FRandRs are indeed valid biomarkers of epileptogenic tissue.

How can this null-result be reconciled with the finding of physiological HFOs reported in other studies? Some studies use cognitive tasks and define as HFOs those oscillations in the HFO frequency band that are modulated by cognitive processing (Axmacher et al., 2008; Kucewicz et al., 2014; Jacobs et al., 2016; Cimbalnik et al., 2018, 2020; Arnulfo et al., 2020; Pail et al., 2020). These findings are in discrepancy with our null result, where we found no evidence for rate modulation of FRandRs by the cognitive tasks. The discrepancy might be reconciled by noting that the absence of evidence does not mean the evidence of absence. In the other studies, subjects performed other tasks. Our data are publicly available and can be tested for physiological HFOs (Boran et al., 2020b, 2019a; Fedele et al., 2020a, 2021). Still, it is conceivable that we recorded physiological FRandRs as well. However, these must have been masked by the consistently high rate of epileptic FRandRs whose overall rate was not modulated in a statistically significant way. This indicates that the number of physiological FRandRs, if at all present, must be small compared to the number of epileptic FRandRs.

### Conclusions

The most important conclusion from our study is that the rate of HFOs, especially the rate of FRandRs, was unaffected by the cognitive tasks. This indicates that the FRandR, our prospective definition of an epileptic HFO, is not confounded by physiological HFOs in the MTL. This is reassuring when using FRandR rate as a biomarker of the EZ.

## Data Availability Statement

The iEEG recordings for two tasks are already publicly available (Boran et al., 2019b, 2020a, 2020b; Fedele et al., 2020a, 2021). The remaining data will be made available after acceptance of the article.

## Code Availability Statement

The code of the HFO detector is freely available at the GitHub repository (https://github.com/ZurichNCH/Automatic-High-Frequency-Oscillation-Detector). The verbal working memory task is available at https://www.neurobs.com/ex_files/expt_view?id=266. The fearful faces video is available in the original AVI format and read by a custom program at https://www.neurobs.com/ex_files/expt_view?id=283.

## Ethics Statement

The studies involving human participants were reviewed and approved by Kantonale Ethikkommission Zürich. The patients/participants provided their written informed consent to participate in this study.

## Author Contributions

JS and EB designed the study. LS treated patients. EB analyzed data and prepared figures and tables. EB and JS wrote the article. All authors critically reviewed the manuscript. All authors contributed to the article and approved the submitted version.

## Conflict of Interest

The authors declare that the research was conducted in the absence of any commercial or financial relationships that could be construed as a potential conflict of interest. The handling Editor is currently organizing a Research Topic with one of the authors JS.

## Footnotes

^1^www.adtechmedical.com
